# Diagnosis of exon 12‐positive polycythemia vera rescued by NGS

**DOI:** 10.1002/ccr3.2720

**Published:** 2020-03-21

**Authors:** Antoine Geay, Bernard Aral, Valentin Bourgeois, Pauline Martin, Fabrice Airaud, Céline Garrec, Stéphane Bézieau, Betty Gardie, François Girodon

**Affiliations:** ^1^ Service d'Hématologie Biologique Pôle Biologie CHU de Dijon Dijon France; ^2^ Laboratoire de génétique chromosomique et moléculaire Pôle Biologie CHU de Dijon Dijon France; ^3^ Service de Génétique Médicale CHU de Nantes Nantes France; ^4^ Ecole Pratique des Hautes Etudes EPHE PSL Research University Nantes France; ^5^ L'institut du Thorax INSERM CNRS UNIV Nantes Nantes France; ^6^ Laboratory of Excellence GR‐Ex Paris France; ^7^ Inserm U1231 Université de Bourgogne Dijon France

**Keywords:** erythrocytosis, Exon 12, JAK2, next generation sequencing, polycythemia vera

## Abstract

A *JAK2*V617F‐negative polycythemia associated with low serum epo needs to be tested for an exon 12 *JAK2* mutation. When negative, due to potential serious complications in PV, a next generation sequencing is necessary to rule out false negative results.

## Graphical abstract

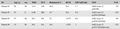

## 1 INTRODUCTION

Nearly 98% of patients with polycythemia vera (PV) harbor a *JAK2* mutation. In these individuals, the mutation is found either on the exon 14 (the classical *JAK2*V617F)^1^ or on exon 12 (in 95% and 3% of cases, respectively).^2^ In a small proportion, atypical mutations of *JAK2* have been reported, including *JAK2*C618R, coexisting *JAK2* V617F/C618R,^3^ and in some exceptional cases a mutation of *CALR*.^4^

Compared with *JAK2* (V617F)‐positive PV patients, those with exon 12 mutations had significantly higher hemoglobin levels and lower platelet and leukocyte counts at diagnosis but similar incidence of thrombosis, myelofibrosis, leukemia, and death.^5^ Moreover, JAK2 exon 12 variation is generally detected at lower level than JAK2V617F.^6^

The exon 12 *JAK2* mutations are variable and heterogeneous including deletions, insertions, and duplications.^7^, ^8^, ^9^ Techniques based on allele‐specific polymerase chain reaction (AS‐PCR) are therefore unsuitable. On the other hand, Sanger sequencing and pyrosequencing do not have sufficient sensitivity for screening in some cases, which is why High Resolution Melting (HRM), a molecular method based on real‐time polymerase chain reaction (PCR), is a method of choice for detecting abnormalities in exon 12 of *JAK2*. HRM analyzes the DNA fusion curves during a gradual increase in temperature, and it appears to be the most efficient current technique for detection of *JAK2* exon 12 mutations^10^ because it is associated with high sensitivity and specificity HRM analysis provides aberrant melting curves indicating the presence of a mutation that must then be characterized using Sanger sequencing. However, reports indicate that cloning was required before sequencing because of low allelic burden in 40% of aberrant melting curves profiles.^11^, ^12^ Indeed, DNA sequencing can overlook mutations with allele frequencies below 15%. Consequently, false negative results are possible with Sanger sequencing even when it is associated with aberrant melting curves.

We report here a series of four patients with low allele burden of *JAK2* exon 12 mutations initially considered as having idiopathic erythrocytosis due to false negative results on *JAK2* exon 12 mutations. The false negatives had been performed using HRM technology but nothing had been observed with Sanger sequencing due to the low allelic burden. We used total blood samples but if we had bone marrow samples, we would have a higher variant allelic frequency.^13^

Over the past 3 years, more than 250 samples from French patients with idiopathic erythrocytosis (defined by either red cell mass > 125% or hematocrit > 60% in male or 56% in female) have been tested using next generation sequencing (NGS) analysis with a dedicated panel including genes involved in the regulation of hypoxia and erythropoietin pathways. Due to the high cost of this technique, patients previously underwent a process of clinical and biological validation in order to rule out obvious causes of polycythemia. The main causes of erythrocytosis had to be excluded in order to restrict the analysis to idiopathic erythrocytosis. In particular, the search for a *JAK2* mutation on exons 12 and 14 had to be negative in order to rule out PV. Surprisingly, in our cohort, in 4 patients (3 men and 1 woman, mean age at diagnosis 65.75 years) low allele burden *JAK2* exon 12 mutations were observed, though they were initially considered negative for this mutation in the local laboratory. We only found 4 patients with low exon 12 mutations and no other ones. Most of them (3/4) had a classical low EPO level and an isolated erythrocytosis. The hematocrit and EPO results of patient #2 are explained because of her age: 97 years old (Table 1). The mutations noted in these patients have been reported many times,^7^ but in our cases, the allele burden was only 6%‐13%, which is below the threshold of sensitivity for Sanger sequencing. Of note, no other mutation (including *LNK, EPOR, HIF, VHL,* or *PHD2*) was observed in the 4 patients.

**Table 1:** Exon 12 mutation and VAF of 4 patients analyzed with NGS method

ID	Age (y)	sex	WBC	PLT	Hematocrit %	RCM	EPO mIU/mL	NGS	VAF
Patient #1	56	M	14.4	330	64.5	NA	1.7	*JAK2* exon 12 p.K539L	8%
Patient #2	97	F	6.40	408	41^a^	NA	9.4	*JAK2* exon 12 p.E543‐D544del	13%
Patient #3	57	M	NA	NA	53	+57%	0.6	*JAK2* exon 12 p.N542‐E543del	6%
Patient #4	53	M	NA	NA	NA	+35%	1	*JAK2* exon 12 p.F537‐K539del‐insL	13%

a The quite low hematocrit rate of patient #2 is explained because she was treated with hydroxyurea for a triple‐negative thrombocythemia when the *JAK2* exon 12 was noted.

Once the presence of *JAK2* exon 12 mutations was detected by NGS analysis, the diagnosis of PV was confirmed and the patients were treated using phlebotomy or cytoreductive therapy to prevent thrombotic events, the most frequent complication in PV. One patient had been experiencing recurrent venous thrombosis and was treated with new oral anticoagulants. If the diagnosis of PV had been made earlier, these thrombotic episodes could have been avoided, underlining the need for an accurate diagnosis, especially when erythrocytosis is associated with low EPO level.^8^ Our results also suggest that aberrant HRM curves should lead to the search for a mutation by Sanger sequencing. Because there is a potential for serious complications, NGS analysis should be recommended when a *JAK2* exon 12 mutation is not detected in order to rule out PV with a low allele burden.

## CONFLICT OF INTEREST

None declared.

## AUTHOR CONTRIBUTIONS

AG and FG: wrote the manuscript; BA, VB, PM, and CG: performed analyses; SB and BG: revised the manuscript.

## References

[1] James C , Delhommeau F , Marzac C , et al. Detection of JAK2 V617F as a first intention diagnostic test for erythrocytosis. Leukemia. 2006;20(2):350‐353. 16341032 10.1038/sj.leu.2404069

[2] Scott LM , Tong W , Levine RL , et al. JAK2 exon 12 mutations in polycythemia vera and idiopathic erythrocytosis. N Engl J Med. 2007;356(5):459‐468. 17267906 10.1056/NEJMoa065202 PMC2873834

[3] Warshawsky I , Mularo F , Hren C , Jakubowski M . Failure of the ipsogen mutascreen kit to detect the *JAK*2 617V>F mutation in samples with additional rare exon 14 mutations: implications for clinical testing and report of a novel 618C>F mutation in addition to 617V>F. Blood. 2010;115(15):3175‐3176. 20395423 10.1182/blood-2009-12-257501

[4] Broséus J , Park J‐H , Carillo S , Hermouet S , Girodon F . Presence of calreticulin mutations in *JAK2*‐negative polycythemia vera. Blood. 2014;124(26):3964‐3966. 25305205 10.1182/blood-2014-06-583161

[5] Passamonti F , Elena C , Schnittger S , et al. Molecular and clinical feature of the myeloproliferative neoplasm associated with *JAK2* exon 12 mutations. Blood. 2011;117(10):2813‐2816. 21224469 10.1182/blood-2010-11-316810

[6] Theocharides A , Passweg JR , Medinger M , et al. The allele burden of JAK2 mutations remains stable over several years in patients with myeloproliferative disorders. Haematologica. 2008;93(12):1890‐1893. 18790796 10.3324/haematol.13074

[7] James C , Ugo V , Le Couédic J‐P , et al. A unique clonal *JAK2* mutation leading to constitutive signalling causes polycythaemia vera. Nature. 2005;434(7037):1144‐1148. 15793561 10.1038/nature03546

[8] Ugo V , Tondeur S , Menot M‐L , et al. Interlaboratory development and validation of a HRM method applied to the detection of *JAK2* Exon 12 mutations in polycythemia vera patients. PLoS ONE. 2010;5(1):e8893. 20126644 10.1371/journal.pone.0008893 PMC2811183

[9] Campbell PJ , Scott LM , Buck G , et al. Definition of subtypes of essential thrombocythaemia and relation to polycythaemia vera based on *JAK2* V617F mutation status. Lancet. 2005;366(9501):1945‐1953. 16325696 10.1016/S0140-6736(05)67785-9

[10] Carillo S , Henry L , Lippert E , et al. Nested high‐resolution melting curve analysis: a highly sensitive, reliable, and simple method for detection of *JAK2* exon 12 mutations – clinical relevance in the monitoring of polycythemia. J Mol Diagn. 2011;13(3):263‐270. 21497288 10.1016/j.jmoldx.2010.12.002 PMC3077738

[11] Rapado I , Grande S , Albizua E , et al. High resolution melting analysis for *JAK2* Exon 14 and Exon 12 mutations. J Mol Diagn. 2009;11(2):155‐161. 19225136 10.2353/jmoldx.2009.080110 PMC2665865

[12] Jones AV , Cross NC , White HE , et al. Rapid identification of *JAK2* Exon 12 mutations using high resolution melting analysis. Haematologica. 2008;93(10):1560‐1564. 18698085 10.3324/haematol.12883

[13] Kjaer L , Westman M , Hasselbalch Riley C , et al. A highly sensitive quantitative real‐time PCR assay for determination of mutant JAK2 Exon 12 allele burden. PLoS ONE. 2012;7(3):e33100. 22403733 10.1371/journal.pone.0033100 PMC3293922

